# Performance of Impulse Oscillometry in Identifying Restrictive Lung Defects in a Veteran Cohort

**DOI:** 10.2174/0118743064304109240611054726

**Published:** 2024-07-19

**Authors:** Danielle R. Glick, Clayton H. Brown, Lan Li, Patricia Gucer, Joanna M. Gaitens, Melissa A. McDiarmid, Stella E. Hines

**Affiliations:** 1 Division of Pulmonary and Critical Care Medicine, School of Medicine, University of Maryland, Baltimore, MD, United States; 2 Department of Veterans Affairs Medical Center, Baltimore, MD, United States; 3 Department of Epidemiology and Public Health, School of Medicine, University of Maryland, Baltimore, MD, United States; 4 Division of Occupational and Environmental Medicine, School of Medicine, University of Maryland, Baltimore, MD, United States

**Keywords:** Impulse oscillometry, Restrictive lung defect, Pulmonary function testing, Spirometry, Lung volumes, Lung function

## Abstract

**Background:**

Impulse oscillometry (IOs) is a technique used to evaluate lung function that uses sound waves imposed over tidal breathing to characterize the airways and lung parenchyma. IOs has been particularly useful in the identification of obstructive lung defects. The present analysis seeks to explore the use of IOs in the identification of restrictive lung physiology among a group of Gulf War I veterans exposed to depleted uranium (DU).

**Methods:**

A total of 36 out of a dynamic 85-veteran cohort attended in-person surveillance visits in 2019 and completed both IOs and PFTs. Performance on IOs was evaluated in a cross-sectional analysis of the group overall and in those identified as having restrictive lung defects defined by either spirometry (FEV1/FVC ≥ LLN and FVC < LLN) or lung volumes (TLC < LLN).

**Results:**

A total of 6 individuals were identified as having restriction (4 based on spirometry alone and an additional 2 by lung volumes). When restriction was present, IOs values of both resistance and reactance were significantly more abnormal.

**Conclusion:**

In the assessment of lung function, IOs may be advantageous over PFTs because it is faster to perform and effort-independent. Although little is known about the utility of IOs in identifying restrictive lung physiology, our results support its use.

## 1 INTRODUCTION

Respiratory impedance measurement is a technique of lung function testing that superimposes sound waves of various frequencies on tidal breathing to characterize the airways and lung parenchyma. Although the technique was first described in the 1950s, its use in clinical practice has been limited due to the lack of robust population reference data and, similarly incomplete understanding of its application [1, 2]. Respiratory impedance testing includes impulse oscillometry (IOs) and forced oscillatory technique (FOT). IOs has distinct advantages over traditional methods of lung function testing, most notably the fact that it is effort-independent and significantly faster to perform [3]. Another advantage is that IOs seems to outperform spirometry in the identification of small airways disease. For example, in a group of individuals exposed to World Trade Center dust who had symptoms of dyspnea and normal spirometry, over half had abnormal IOs in a pattern suggestive of small airways disease [4].

Our group previously reported similar findings in a group of Gulf War I veterans exposed to depleted uranium (DU) inhalation during a series of friendly fire incidents [5]. Since 1993, a subset of those veterans has undergone biennial in-person medical surveillance visits at the Baltimore VA Medical Center [6]. During these visits, participants completed a comprehensive health assess-ment including a full history and physical, several health questionnaires, and lung function testing with the goal of evaluating the effect of DU exposure on the veterans’ health. Potential for DU-related respiratory toxicity exists related to inhalation of DU oxides at the time of exposure, as well as retention of DU in regional lymph nodes systemically [7-9]. To date, our group has not identified any DU-related effect on the pulmonary health of this cohort. Cross-sectional analysis of spirometry since 1999 has consistently been normal, and longitudinal spirometry data over 20 years demonstrate a rate of decline in lung function that is comparable to the general population [10, 11]. In 2015, to better assess the presence of small airway dysfunction, IOs testing was added to the surveillance battery [5]. Over two consecutive assessments, IOs consistently identified a higher frequency of participants defined as obstructed when compared with traditional spirometry. Neither of these efforts, however, attempted to characterize restrictive lung disease by IOs outcomes.

Conventional assessment of the mechanical properties of the lungs parses pathophysiology into obstructive and restrictive defects. Using these concepts, airflow obstruction easily correlates with airflow resistance on IOs [3]. IOs reactance values are thought to reflect the capacitance or stiffness of the lungs. Reactance, then, should correlate with restrictive defects [3]. Diffuse parenchymal or interstitial lung diseases are commonly characterized by restrictive defects on pulmonary physiology testing. Restrictive defects are diagnosed using lung volume measurement and may be suggested by certain abnormalities on spirometry. Lung volume measurement can be cumbersome and require specialized, unwieldy, non-mobile equipment. Spirometry uses more convenient equipment but depends on participant effort provider coaching and may take significant time if acceptable and repeatable maneuvers are not easily obtained. Alternatively, respiratory impedance testing can be quickly performed, requires minimal patient effort, and can be portable [3, 12]. If IOs can identify abnormalities associated with restrictive defects, it could serve as an additional tool in the evaluation of interstitial lung disease. Previous work on small cohorts examining respiratory impedance testing in interstitial lung disease has shown that both resistance and reactance tend to be abnormal when compared to normal controls [13, 14].

We sought to identify whether abnormal IOs might be useful in identifying restrictive lung defects. Using our DU population as a convenience sample, we hypothesized that IOs values would be abnormal when a pattern of restriction is present based on traditional definitions by spirometry and lung volume testing. As a secondary objective, we evaluated whether patients with higher DU body burden would be more likely to have restrictive pulmonary defects, either by lung volume measurement or by spirometry.

## 2 MATERIALS AND METHODS

### 2.1 Setting

During the months of March through June of 2019, 36 veterans attended in-person visits at the Baltimore Veterans Affairs Medical Center. During three days, veterans completed health history questionnaires, urine uranium testing, full pulmonary function testing, and IOs, in addition to other non-pulmonary evaluations [15]. Informed consent and institutional review board approval were obtained as described previously [15].

### 2.2 Testing

IOs measurements were obtained using a SensorMedics Carefusion Vmax™ system (Yorba Linda, CA) according to system protocols. Testing was performed by respiratory therapists who were certified by the National Board for Respiratory Care. Measurements of interest for IOs included resistance at 5 Hz (R_5_), resistance at 20 Hz (R_20_), frequency dependence of resistance (R_5-20_), reactance at 5 Hz (X_5_), area of reactance (AX), and resonant frequency (Fres). Values for percent predicted of normal for IOs resistance values were as per Vogel [16]. All testing was done without bronchodilator administration, with IOs testing preceding pulmonary function testing.

Pulmonary function testing, including spirometry, body plethysmography, and diffusion capacity measurement, was performed using the Morgan Scientific system (Haverhill, MA) according to the American Thoracic Society guidelines [17, 18]. The following measurements of interest were obtained: Spirometry - forced expiratory volume in 1 second (FEV1), forced vital capacity (FVC), the ratio of FEV1/FVC, and forced expiratory flow at 25-75% vital capacity (FEF_25-75%_); Lung volumes: total lung capacity (TLC), functional residual capacity (FRC), residual volume (RV), the ratio of RV/TLC; and diffusion capacity (DLCO).

### 2.3 Data Analysis

Outcome variables (spirometry, lung volumes, diffusion capacity, and oscillometry measures) are presented as means with standard deviations both in the group overall, as stratified by DU body burden level, and by the prevalence of restrictive lung disease pattern. Other variables of interest (age, height, weight, race, and smoking) are presented similarly. For comparison of differences between both the high and low DU groups and between the restriction groups, either the Fisher’s or Mann-Whitney tests were employed as appropriate. We reported the means and standard deviations of outcome variables as they generally did not have skewed distributions.

DU body burden was dichotomized around the value of 0.1 µg U/g Cr, as has been previously described in this cohort [10]. This cutpoint is between the NHANES 95% value in non-exposed individuals (0.034 µg U/g creatinine) and the upper limit of normal for individuals in areas with naturally elevated levels of U in food and water (0.35 µg U/g creatinine) [9]. In our population, this cut-point has historically correlated with the presence of a retained DU-containing fragment [10]. Smoking exposure was captured as either “never”, “ever”, or “current”.

The presence of restriction was explored using three different definitions of restriction based on either spirometry or lung volumes. Using spirometry, the restriction was defined as an FEV1/FVC greater than or equal to the lower limit of normal (LLN) and an FVC less than the lower limit of normal as has been used in the evaluation of other cohorts exposed to occupational or environmental hazards [19, 20]. We also used two definitions of restriction based on lung volume measurements – 1) TLC less than or equal to the lower limit of normal or 2) TLC less than or equal to the lower limit of normal, RV less than or equal to the lower limit of normal, and FVC less than or equal to the lower limit of normal. The second definition was too selective, and none of our participants met these criteria. As such, only the first definition and the spirometry definition of restriction were used.

Finally, we explored the association between outcomes on IOs and PFTs with Pearson correlations. Means and comparison analyses were performed using SAS; correlations were performed using SPSS version 20.

## 3 RESULTS

The 36 participants were stratified by the presence of restriction based on the two previously described methods. Demographics of the population are described in Table **1**.

All participants were male. Age, height, weight, and smoking status were not significantly different between those defined as restricted *vs* not. While there was not a significant difference between restriction groups with respect to BMI and race, it is worth noting that BMI was higher in the restricted group (35.52 *vs* 31.69 kg/m^2^), and a greater proportion of those classified as restricted were African American (66.7% *vs* 23.3%). There was no significant difference in age, height, weight, BMI, race, or smoking status between those with low *vs* high uranium (Appendix). There was also no difference in the prevalence of either spirometric or lung-volume-based restriction based on DU exposure status, as displayed in Table **2**.

Table **3** contains the PFT and IOs data for the group overall and as stratified by restrictive physiology. When stratified by restrictive pattern, all IOs outcomes were significantly different between the two groups. Among the pulmonary function tests, significant between-group differences were found for forced expiratory volume in 1 second (FEV1), forced vital capacity (FVC), total lung capacity (TLC), functional residual capacity (FRC), and residual volume (RV). The remainder of the tests (FEV1/FVC, mid-expiratory flow, flow FEF_25-75%_, RV/TLC, and diffusion capacity, DLCO) were not different between the restricted and not restricted groups. With respect to DU exposure status, there was no significant difference in performance on either testing modality between the low *vs* high uranium groups, although in general, the high uranium group performed better on these tests (Appendix).

Correlations between IOs and PFT variables in the group overall are presented in Table **4**. This demonstrates strong correlations between FEV1 and FVC and all IOs outcomes, as well as a significant relationship between TLC and most IOs outcomes. FEF_25-75%_ and RV/TLC correlated with IOs outcomes of reactance but not resistance, while FRC correlated only with R_5_, R_20_, and Fres. No significant correlations were found between FEV1/FVC, RV, and DLCO and IOs outcomes.

## 4 DISCUSSION

We sought to identify whether abnormal IOs might be useful in the identification of restrictive lung defects. In this veteran cohort, restrictive defects defined by traditional testing were associated with multiple IOs parameters. Veterans with restrictive physiology by lung volumes had IOs values that crossed thresholds of abnormality for all parameters tested. Veterans without restrictive physiology had normal values on IOs testing. These findings seem to suggest that IOs testing could help identify patients with restrictive pulmonary physiology without the need for cumbersome lung volume measurement. IOs may be easier to administer while still able to identify patients for whom additional diagnostic evaluation is warranted.

Previous work has shown IOs to be valuable in identifying obstructive lung disease, not only COPD and asthma, but also potentially otherwise unrecognized small airways disease [21]. In restrictive lung states, however, relatively little is known about the performance of IOs. Early work showed that resistance values may be normal in the presence of diffuse pulmonary disease or that interstitial lung diseases may have abnormalities of both resistance and reactance in a pattern indistinguishable from obstructive lung disease [13, 14]. There has been a newer focus on the use of intra-breath variability of reactance as a method of distinguishing restrictive and obstructive defects. This is done by measuring the reactance during both inspiration and expiration and observing them separately, which was outside of the scope of data collection for our study. Two recent studies found that the within-breath variance of X_5_ successfully differentiates between interstitial lung disease and COPD [22, 23]. It was suggested that this may reflect the difference in elastic lung recoil present in each disease state.

Using IOs as an adjunct or even in place of PFTs requires further understanding of the expected patterns of abnormalities. In a similar cohort of symptomatic deployers, Butzko *et al*. previously demonstrated an association of abnormalities in AX and R_4-20_ on forced oscillatory technique (similar to IOs) with reductions in FEV_1_, FVC, and FEF_25-75_ [24]. Our findings also suggest that certain traditional PFT parameters correlate with IOs outcomes. For instance, resistance values (R_5_, R_20_, and R_5-20_) strongly correlated with FEV1 and FVC, which was expected. Less strong but still significant relationships were found between reactance values (X_5_, AX, and Fres) and TLC, lending support to the idea that reactance reflects the capacitance and stiffness of the lung in the traditional way restriction is understood. Of note, this relationship was also observed between R_20_ and R_5-20_, again underscoring that overall abnormal IOs may reflect restrictive states.

Of interest in this population of DU-exposed veterans is the fact that there was no significant difference in performance on PFTs or IOs between those with high *vs* low urine uranium levels. Similarly reassuring is the fact that performance on both tests was, on average, within normal limits for the cohort overall. This is consistent with previous work done by our group [5, 10, 11].

This study is limited by a small sample size of all male participants. The observations, especially within the group defined as restricted, need to be repeated in our cohort to establish the persistence of the findings as well as reproduction in a larger group. Although spirometry is more accessible and easier to perform, it may falsely misclassify restriction, particularly among patients with significant air trapping, and lung volume measurement remains the gold standard for assessing restrictive pulmonary physiology [25]. Ideally, we would have examined any differences in IOs outcomes between the groups, but as there were only a total of 6 participants defined as restricted, further subclassification would not have been statistically meaningful. Despite this, the fact that IOs values were significantly different when a restrictive pattern was present based on traditional definitions is noteworthy. In addition to replication on a larger scale, exploration of whether a specific phenotype (*i.e*., pattern of abnormality) on IOs testing is present when restrictive physiology exists would significantly broaden the potential clinical applications of IOs.

## CONCLUSION

In summary, we have shown that in a small sample of veterans, patterns of restrictive lung defects were associated with overall abnormal IOs values. IOs may serve a role as an easier, more efficient screening tool for detecting restriction. While there was not a specific phenotype of IOs abnormality associated with restriction, the correlation of IOs with PFT parameters suggests that in a larger group, stronger patterns may emerge. At present, IOs offers a useful adjunct to traditional lung function testing and may unearth otherwise unidentified abnormalities. Given the ease of use and portability of some IOs testing devices, it could potentially have broader applications in public health, including medical surveillance for occupational and exposure-related lung diseases. Moreover, there is a need for larger, population-based studies to both establish more robust reference values for IOs and to further explore the performance of IOs in comparison to the current gold standard of pulmonary function testing.

## AUTHORS’ CONTRIBUTIONS

Conceptualization was presented by D.G. and S.H.; methodology was given by P.G., C.B., and L.L.; software was curated by C.B. and L.L.; validation was done by D.G. and C.B..; formal analysis was done by C.B. and L.L.; investigation was conducted by D.G., J.G., M.M., and S.H.; resources were retrieved by M.M. and S.H.; data curation was performed by D.G. and L.L.; the original draft was prepared by D.G.; review and editing were done by C.B., J.G., M.M., and S.H.; visualization was done by D.G.; supervision was provided by S.H.; project administration was contributed by M.M.; funding acquisition was completed by M.M. All authors read and agreed to the published version of the manuscript.

## Figures and Tables

**Appendix:** Demographics and pulmonary function testing in the group overall and as stratified by DU exposure status.

-	Overall (n=36)	Low DU (n = 26)	High DU (n = 10)	p-value
-	Mean (SD)			-
Age (years)	53.1 (4.9)	53.2 (5.3)	52.7 (4.0)	0.93^†^
Height (cm)	179.0 (7.3)	178.3 (8.2)	180.6 (4.3)	0.433^†^
Weight (kg)	103.4 (21.6)	104.8 (21.8)	99.9 (21.7)	0.639^†^
BMI (kg/m^2^)	32.3 (6.7)	33.0 (6.7)	30.7 (6.8)	0.349^†^
	N(%)	N (%)		
Race, African American	11 (31%)	7 (27%)	4 (40%)	0.454‡
Smoking: Current Ever	10 (28%) 18 (50%)	6 (23%) 12 (46%)	4 (40%) 6 (60%)	0.413‡ 0.711‡
**Impulse Oscillometry**				
R_5_, kPa/L/sec	0.36 (0.12)	0.37 (0.13)	0.33 (0.09)	0.513
R_5_, (% predicted)	125.1 (42.3)	128.7 (45.7)	115.8 (32.4)	0.454
R_20_, kPa/L/sec	0.28 (0.07)	0.29 (0.08)	0.27 (0.06)	0.646
R_20_ (% predicted)	114.0 (30.1)	115.9 (32.6)	109.1 (23.0)	0.768
R_5-20_, %	19.1 (10.7)	19.1 (10.7)	19.9 (10.7)	0.543
X_5_, kPa/L/sec	-0.11 (0.07)	-0.11 (0.1)	-0.10 (0.0)	0.986
AX, kPa/L	0.61 (0.65)	0.61 (0.7)	0.69 (0.7)	0.614
Fres, Hz	14.8 (4.2)	15.1 (4.5)	13.8 (3.3)	0.475
**Pulmonary Function Testing**				
FEV1, L	3.58 (0.66)	3.49 (0.70)	3.83 (0.46)	0.074
FVC, L	4.66 (0.87)	4.53 (0.90)	4.99 (0.73)	0.145
FEV1/FVC, %	77.2 (6.7)	77.1 (7.1)	77.2 (6.0)	0.794
FEF_25-75%_, L	3.32 (1.1)	3.26 (1.17)	3.48 (0.89)	0.741
TLC, L	6.87 (1.27)	6.78 (1.32)	7.10 (1.16)	0.497
FRC, L	3.31 (0.92)	3.29 (1.02)	3.37 (0.62)	0.614
RV, L	2.12 (0.63)	2.15 (0.64)	2.06 (0.64)	0.986
RV/TLC, %	30.64 (6.15)	31.4 (6.0)	28.6 (6.5)	0.520
DLCO, mL/min/mmHg	28.2 (4.5)	27.8 (5.0)	29.4 (2.4)	0.286

**Note:** ^†^Mann-Whitney U-test, ‡Fisher’s test. BMI, body mass index. R_5_ and R_20_ – resistance at 5 and 20 Hz, respectively (normal <150%)^20^; kPa – kilopascals; R_5-20_ – frequency dependence of resistance (normal <20-30%)^21^; X_5_ – reactance at 5 Hz (normal >-0.1176 kPa/L/s)^4^; AX – area of reactance (normal <0.33 kPa/L/s)^3^; Fres – resonant frequency (normal <12 Hz)^20^. FEV1 – forced expiratory volume at 1 second; FVC – forced vital capacity; FEF_25-75%_ - forced expiratory flow between 25-75%; TLC – total lung capacity; FRC – functional residual capacity; RV – residual volume; DLCO – diffusing capacity of carbon monoxide. *p<0.05, **p<0.01, ***p<0.001.

**Table 1:** Demographic characteristics of the veteran cohort overall and as stratified by pulmonary restriction status.

-	Overall (n = 36)	Restricted Group (n=6)	Not Restricted group (n=30)	p-value
-	**Mean (SD)**	**Mean (SD)**		-
Age (years)	53.1 (4.9)	52.5 (1.9)	53.2 (5.3)	0.815^†^
Height (cm)	179.0 (7.3)	180.5 (6.9)	178.7 (7.5)	0.535^†^
Weight (kg)	103.4 (21.6)	115.5 (17.4)	101.0 (21.8)	0.098^†^
BMI (kg/m^2^)	32.3 (6.7)	35.52 (5.4)	31.69 (6.8)	0.111^†^
	**N(%)**	**N(%)**		
Race, African American	11 (31%)	4 (66.7%)	7 (23.3%)	0.057‡
Smoking: Current Ever	10 (28%) 18 (50%)	0 (0%) 2 (33%)	10 (33%) 16 (53%)	0.157‡ 0.658‡

**Note:** ^†^Mann-Whitney U-test, ‡Fisher’s test. BMI, body mass index.

**Table 2:** Prevalence of restriction based on two traditional definitions and DU exposure status.

Definition of Restriction	Low DU (n = 26)	High DU (n = 10)	Fisher’s p-value
Spirometry: FEV1/FVC ≥ LLN AND FVC < LLN	3 (11.54)	1 (10.00)	0.992
Lung Volumes: TLC < LLN	4 (15.38)	2 (20.00)	0.764

**Note: **FEV1/FVC – the ratio of forced expiratory volume in 1 second (FEV1) to forced vital capacity (FVC). LLN – lower limit of normal. TLC – total lung capacity.

**Table 3:** Pulmonary function testing in the group overall and as stratified by pulmonary restriction status.

-	Overall (n=36)	Restricted Group (n=6)	Not Restricted Group (n=30)	p-value
-	Mean (SD)			-
**Impulse Oscillometry**				
R_5_, kPa/L/sec	0.36 (0.12)	0.5 (0.15)	0.33 (0.09)	0.013*
R_5_, (% predicted)	125.1 (42.3)	174.83 (52.4)	115.17 (32.81)	0.014*
R_20_, kPa/L/sec	0.28 (0.07)	0.35 (0.08)	0.27 (0.06)	0.023*
R_20_ (% predicted)	114.0 (30.1)	141.5 (34.58)	108.53 (26.44)	0.026*
R_5-20_, %	19.1 (10.7)	28.9 (5.66)	17.17 (10.4)	0.015*
X_5_, kPa/L/sec	-0.11 (0.07)	-0.19 (0.12)	-0.09 (0.04)	0.005**
AX, kPa/L	0.61 (0.65)	1.45 (1)	0.45 (0.41)	0.004**
Fres, Hz	14.8 (4.2)	19.69 (3.51)	13.76 (3.6)	0.002**
**Pulmonary Function Testing**				
FEV1, L	3.58 (0.66)	2.85 (0.5)	3.73 (0.59)	0.004**
FVC, L	4.66 (0.87)	3.57 (0.42)	4.88 (0.77)	0.001**
FEV1/FVC, %	77.2 (6.7)	79.5 (8.41)	76.7 (6.39)	0.242
FEF_25-75%_, L	3.32 (1.1)	3.01 (1.24)	3.38 (1.08)	0.596
TLC, L	6.87 (1.27)	5.2 (0.49)	7.2 (1.1)	0.0001***
FRC, L	3.31 (0.92)	2.48 (0.46)	3.48 (0.89)	0.012*
RV, L	2.12 (0.63)	1.54 (0.52)	2.24 (0.59)	0.023*
RV/TLC, %	30.64 (6.15)	29.17 (8.16)	30.93 (5.79)	0.670
DLCO, mL/min/mmHg	28.2 (4.5)	27.24 (3.59)	28.45 (4.68)	0.511

**Note: **R_5_ and R_20_ – resistance at 5 and 20 Hz, respectively (normal <150%)^20^; kPa – kilopascals; R_5-20_ – frequency dependence of resistance (normal <20-30%)^21^; X_5_ – reactance at 5 Hz (normal >-0.1176 kPa/L/s)^4^; AX – area of reactance (normal <0.33 kPa/L/s)^3^; Fres – resonant frequency (normal <12 Hz)^20^. FEV1 – forced expiratory volume at 1 second; FVC – forced vital capacity; FEF_25-75%_ - forced expiratory flow between 25-75%; TLC – total lung capacity; FRC – functional residual capacity; RV – residual volume; DLCO – diffusing capacity of carbon monoxide. *p<0.05, **p<0.01, ***p<0.001.

**Table 4:** Correlation (pearson’s test) of IOs and PFT values.

-	**FEV1**	**FVC**	**FEV1/FVC**	**FEF_25-75_**	**TLC**	**RV**	**RV/TLC**	**FRC**	**DLCO**
**R_5_**	-0.596***	-0.556***	-0.071	-0.319	-0.438	-0.124	0.188	-0.363*	-0.307
**R_20_**	-0.501**	-0.477**	-0.028	-0.290	-0.399*	-0.158	0.106	-0.381*	-0.250
**R_5-20_**	-0.464**	-0.450**	-0.032	-0.150	-0.337*	-0.043	0.217	-0.249	-0.232
**X_5_**	0.632***	0.565***	0.188	0.408*	0.373*	0.017	-0.356*	0.230	-0.304
**AX**	-0.657***	-0.572***	-0.207	-0.420*	-0.348*	0.057	0.406*	-0.169	-0.316
**Fres**	-0.703***	-0.667***	-0.069	-0.352*	-0.476**	-0.036	0.357*	-0.352*	-0.328

Note: *p<0.05, **p<0.01, ***p<0.001.

## Data Availability

The source of data are the surveillance results of this DU exposed cohort.

## LIST OF ABBREVIATIONS

IOs: Impulse oscillometry
DU: Depleted Uranium
FOT: Forced Oscillatory Technique
FV: Forced Vital Capacity
TLC: Total Lung Capacity
FRC: Functional Residual Capacity
RV: Residual Volume

## STANDARDS OF REPORTING

STROBE guidelines were followed.
